# Exosomes in Skin of Color: Dermatological Insights, Therapeutic Potential, and Clinical Realities in India

**DOI:** 10.7759/cureus.102145

**Published:** 2026-01-23

**Authors:** Kajomi Shingala, Dipesh Nariya

**Affiliations:** 1 Department of Dermatology, Shri M. P. Shah Government Medical College, Jamnagar, IND; 2 Department of Pharmacology, Shri M. P. Shah Government Medical College, Jamnagar, IND

**Keywords:** acne scars, alopecia, dermatology, exosomes, extracellular vesicles, india, pigmentation disorders, regenerative medicine, skin of color

## Abstract

Exosomes are nanosized extracellular vesicles that mediate intercellular communication by transferring proteins, lipids, and nucleic acids, and growing experimental evidence indicates their involvement in key cutaneous processes such as inflammation, melanogenesis, wound repair, angiogenesis, fibroblast activation, and hair follicle cycling. These biological properties have generated substantial interest in the use of exosome-based therapies in regenerative and aesthetic dermatology, although clinical adoption has often outpaced the strength of supporting evidence and regulatory oversight - particularly in India, where skin-of-color considerations, high disease burden, and rapid commercialization converge.

This narrative review synthesizes current understanding of exosome biology, therapeutic potential, clinical evidence, and safety considerations in dermatology, with emphasis on pigmentary disorders, acne scarring, hair disorders, wound healing, and inflammatory dermatoses relevant to Indian practice. Preclinical studies suggest that mesenchymal stem cell- and skin-derived exosomes can modulate immune signaling, melanocyte function, angiogenesis, and extracellular matrix remodeling, while early clinical reports indicate possible benefits in pigmentation, scar modulation, hair loss, and tissue repair. However, available human data are largely derived from small, uncontrolled studies employing heterogeneous products and protocols, with limited representation of skin-of-color populations and insufficient long-term safety data. In the Indian context, gaps in product standardization, quality control, regulatory clarity, and informed consent further complicate clinical translation. While exosomes represent a promising frontier in dermatology, their use should presently be considered investigational and approached with caution, transparency, and robust ethical safeguards, until standardized manufacturing processes, population-specific trials, and comprehensive safety data become available.

## Introduction and background

The field of dermatology has witnessed a paradigm shift over the past decade, moving beyond conventional pharmacologic interventions toward regenerative, cell-free, and biologically inspired therapies. Among these emerging modalities, exosomes - nanosized extracellular vesicles (EVs) involved in intercellular communication - have generated significant scientific and clinical interest. Initially characterized as cellular waste products, exosomes are now recognized as critical mediators of physiological and pathological signaling, capable of modulating immune responses, tissue repair, angiogenesis, and melanocyte function [1,2].

Exosomes are lipid bilayer-enclosed vesicles, typically 30-150 nm in diameter, released by almost all cell types and present in various biological fluids. Their molecular cargo includes proteins, lipids, messenger RNA, microRNA, and other non-coding RNAs, enabling them to influence recipient cells in a targeted and context-dependent manner [3]. In dermatologic biology, exosomes participate in epidermal-dermal crosstalk, immune surveillance, pigmentation pathways, and hair follicle cycling - processes central to both inflammatory and aesthetic skin disorders [4].

Preclinical studies have demonstrated that exosomes derived from mesenchymal stem cells (MSCs), fibroblasts, keratinocytes, and immune cells can modulate key pathways involved in wound healing, fibrosis, inflammation, and melanogenesis [5-7]. These findings have catalyzed growing interest in exosome-based interventions for a wide range of dermatologic conditions, including acne scarring, photoaging, pigmentary disorders, alopecia, and chronic inflammatory dermatoses. Importantly, exosomes offer theoretical advantages over cell-based therapies, including lower immunogenicity, absence of replicative potential, and greater biological stability, positioning them as attractive candidates for translational applications [8].

However, the rapid expansion of exosome-based therapies into clinical dermatology has outpaced the generation of high-quality clinical evidence. While early-phase studies and experimental models suggest therapeutic potential, robust randomized controlled trials remain limited. This disparity between scientific promise and clinical validation has been particularly pronounced in aesthetic and regenerative dermatology, where commercial adoption often precedes regulatory clarity and consensus guidelines [9].

The Indian dermatology landscape presents a uniquely complex context for evaluating exosome-based therapies. India bears a high burden of pigmentary disorders, acne sequelae, hair loss, and scarring, particularly among individuals with Fitzpatrick skin types IV-VI [10]. These conditions are often chronic, psychologically distressing, and therapeutically challenging, driving both patients and clinicians to explore novel interventions. Concurrently, India has witnessed a rapid rise in aesthetic dermatology clinics offering exosome-based treatments, frequently marketed as “stem cell-derived” or “regenerative” solutions, despite limited standardization and variable supporting evidence.

Regulatory oversight of exosome-based products in India remains evolving. Exosomes occupy a grey zone between biologics, cell-derived products, and cosmeceuticals, leading to heterogeneity in manufacturing practices, quality control, and clinical claims [11]. Unlike conventional pharmaceuticals, many exosome formulations used in clinical practice lack uniform characterization, standardized dosing, or long-term safety data. This regulatory ambiguity raises important ethical and medico-legal considerations, particularly in a setting where direct-to-consumer marketing and social media influence are increasingly prominent.

From a scientific standpoint, extrapolation of global data to Indian patients requires careful consideration. Skin of color exhibits distinct biological behavior with respect to inflammation, pigmentation, and wound healing. Pathways modulated by exosomes - such as transforming growth factor-β (TGF-β) signaling, melanocyte-keratinocyte interactions, and inflammatory cytokine networks - may differ in expression and clinical relevance across ethnic populations [10]. Consequently, therapeutic outcomes observed in predominantly Western cohorts may not reliably predict efficacy or safety in Indian patients. Despite these challenges, exosome-based therapies represent a potentially transformative frontier in dermatology if developed and applied judiciously. A balanced appraisal that integrates mechanistic insights, available clinical evidence, regulatory realities, and ethical practice considerations is essential to guide their responsible adoption.

This review aims to critically examine the role of exosomes in dermatology, with a particular focus on their translational relevance and clinical applicability in the Indian context. We synthesize current evidence on exosome biology, therapeutic mechanisms, and dermatologic indications, while highlighting gaps in evidence, challenges in standardization, and unmet research needs specific to Indian practice. By contextualizing global advances within regional realities, this article seeks to provide dermatologists with a scientifically grounded framework for evaluating the promise and limitations of exosome-based therapies in contemporary dermatology.

## Review

Biology of exosomes and mechanisms relevant to skin

Exosomes are a subclass of EVs originating from the endosomal compartment and released into the extracellular milieu through fusion of multivesicular bodies with the plasma membrane. Their biogenesis is a tightly regulated process involving endosomal sorting complexes required for transport (ESCRT)-dependent and ESCRT-independent pathways, which govern cargo selection and vesicle release [12]. This regulated packaging distinguishes exosomes from other EVs and underpins their functional specificity in tissue homeostasis and disease.

**Figure 1:**
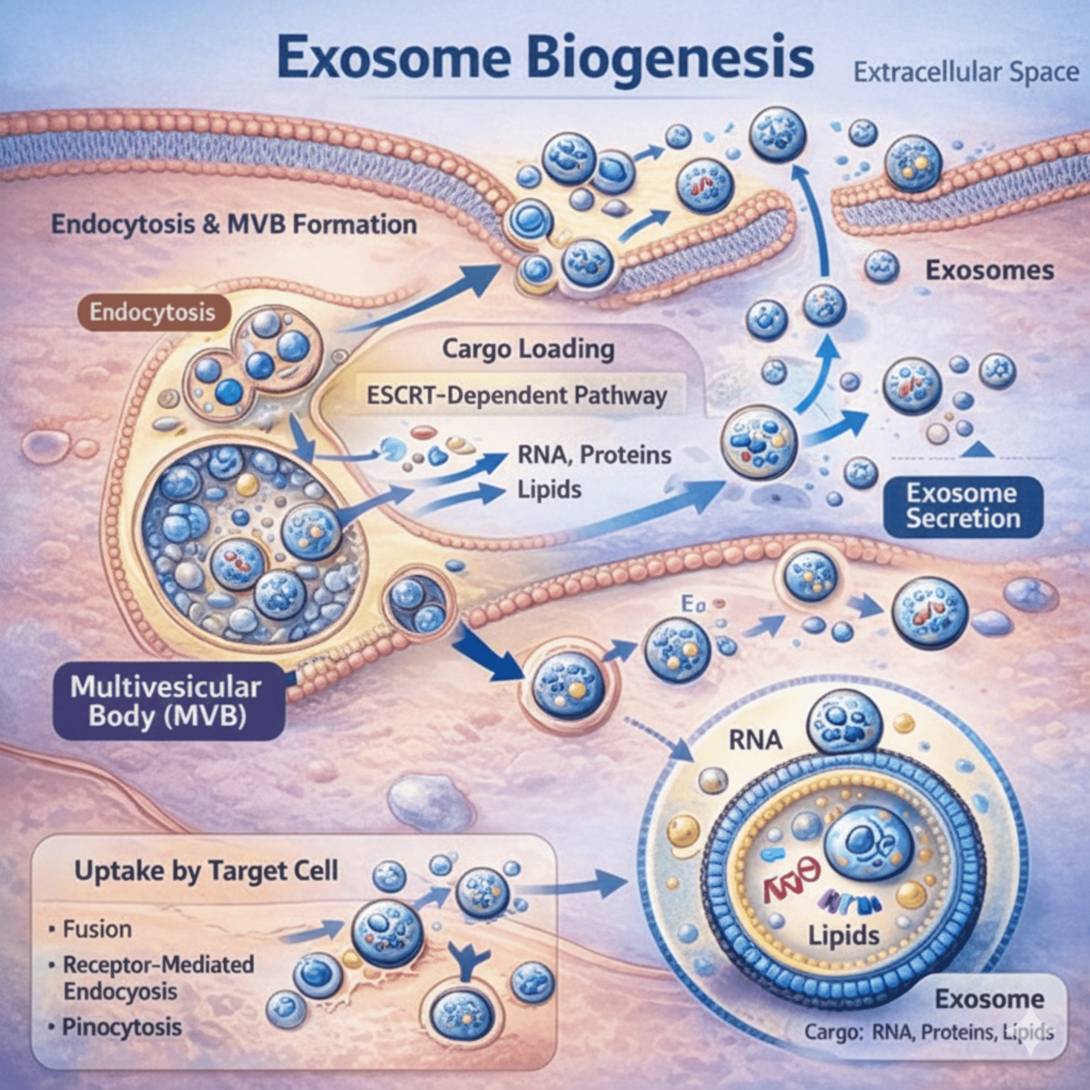
Exosome biogenesis and secretion pathway Schematic illustration of exosome formation within the endosomal system. Early endosomes mature into multivesicular bodies (MVBs), where intraluminal vesicles form and selectively incorporate RNA, proteins, and lipids via endosomal sorting complex required for transport (ESCRT)-dependent and ESCRT-independent mechanisms. Fusion of the MVB with the plasma membrane results in the release of exosomes into the extracellular space. Exosomes may then be taken up by recipient cells through membrane fusion, receptor-mediated endocytosis, or pinocytosis, enabling the transfer of functional molecular cargo. This is an original illustration.

In the skin, exosomes serve as critical mediators of intercellular communication between keratinocytes, melanocytes, fibroblasts, immune cells, and endothelial cells. By transferring bioactive molecules such as microRNAs, cytokines, growth factors, and signaling lipids, exosomes influence cellular proliferation, differentiation, migration, and immune modulation - processes fundamental to epidermal turnover, wound repair, pigmentation, and inflammatory responses [13]. Figure 1 presents an illustration of the exosome biogenesis and secretion pathway.

Keratinocyte-derived exosomes play an important role in epidermal-dermal crosstalk by modulating fibroblast activity and extracellular matrix remodeling. Experimental models have demonstrated that these exosomes regulate collagen synthesis and matrix metalloproteinase expression, thereby influencing scar formation and photoaging-related changes [14]. Similarly, fibroblast-derived exosomes contribute to dermal homeostasis by promoting keratinocyte migration and re-epithelialization during wound healing.

Melanocyte-keratinocyte communication, a key determinant of pigmentation biology, is also mediated in part by exosomes. Exosomal transfer of microRNAs and signaling molecules has been shown to regulate melanogenesis, melanosome transport, and pigment distribution within the epidermis. Dysregulation of these pathways may contribute to pigmentary disorders such as melasma and post-inflammatory hyperpigmentation, conditions that are particularly prevalent and therapeutically challenging in Indian populations [15].

Exosomes derived from MSCs have received the greatest attention in dermatologic research due to their potent immunomodulatory and regenerative properties. MSC-derived exosomes have been shown to suppress pro-inflammatory cytokine production, promote angiogenesis, and enhance fibroblast and keratinocyte proliferation through activation of pathways such as PI3K/Akt, Wnt/β-catenin, and TGF-β signaling [16,17]. These effects collectively contribute to improved wound healing and tissue regeneration in preclinical models.

**Figure 2:**
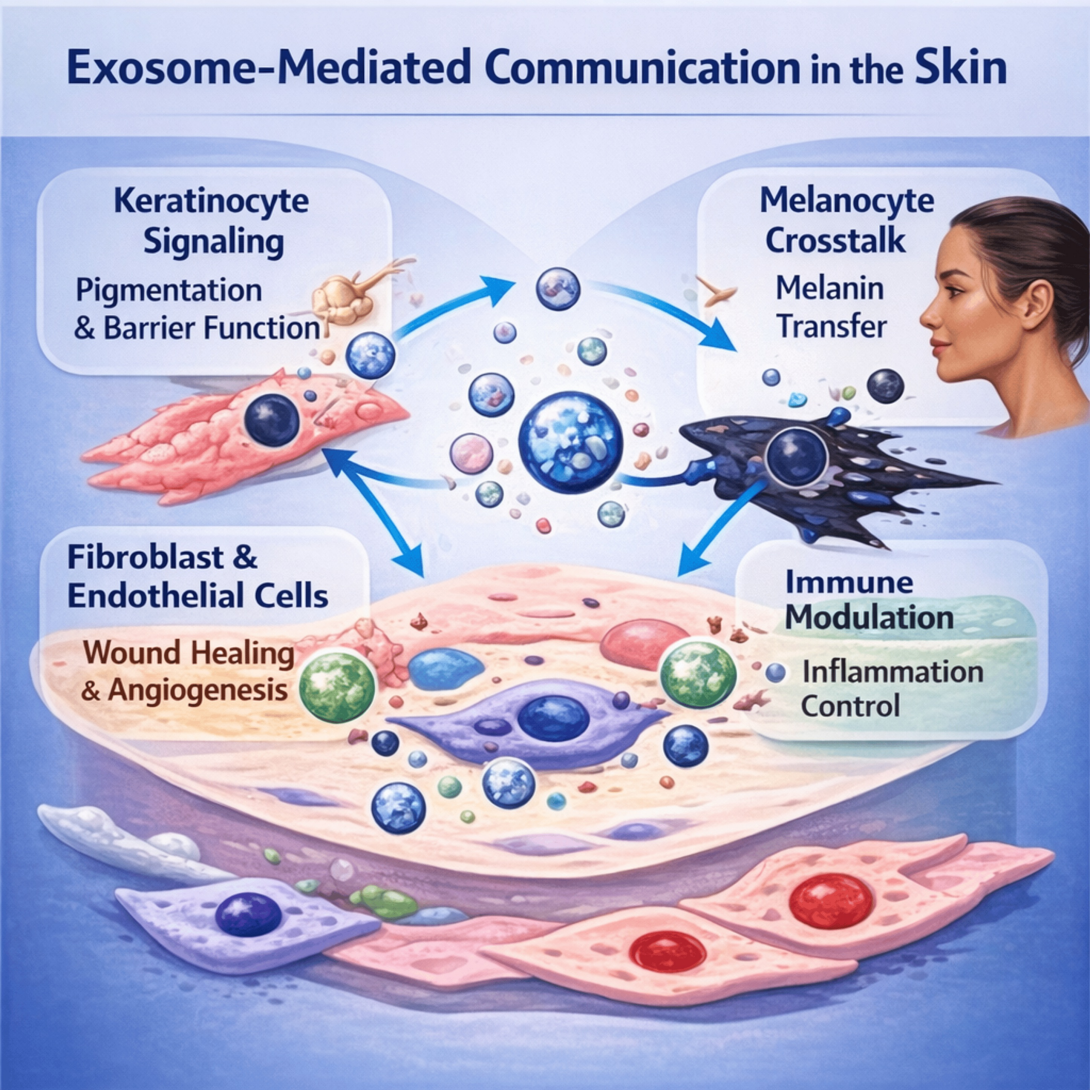
Exosome-mediated intercellular communication in the skin Overview of exosome-driven signaling networks in cutaneous tissue. Keratinocyte-derived exosomes influence pigmentation pathways and barrier function; melanocyte exosomes participate in pigment transfer; fibroblast and endothelial exosomes promote wound healing and angiogenesis; and immune-cell-derived vesicles modulate inflammatory signaling. Together, these vesicle-mediated interactions contribute to pigmentation control, immune homeostasis, tissue repair, and extracellular matrix regulation. This is an original illustration.

Immune modulation represents another key mechanism by which exosomes influence skin disease. Exosomes can alter antigen presentation, T-cell activation, and macrophage polarization, thereby shaping local inflammatory responses. In inflammatory dermatoses, exosomal cargo may either propagate or suppress inflammation, depending on the cellular source and microenvironmental context. This duality underscores the complexity of exosome biology and the need for precise characterization when considering therapeutic applications [18]. Figure 2 presents exosome-mediated intercellular communication in the skin.

From a translational perspective, the biological activity of exosomes is highly dependent on their cellular origin, isolation method, and cargo composition. Variability in these parameters can lead to significant differences in functional outcomes, even among exosomes derived from the same cell type. This heterogeneity poses challenges for standardization, reproducibility, and clinical translation - issues that are particularly relevant in settings where exosome products are introduced into clinical practice without uniform manufacturing oversight [19]. Table 1 presents the biological roles of exosomes in skin.

**Table 1:** Biological roles of exosomes in skin miRNAs: micro RNAs; ECM: extracellular matrix; MMP: matrix metalloproteases; PDGF: platelet-derived growth factor; VEGF: vascular endothelial growth factor; TGF: transforming growth factor

Source cell	Key cargo	Target cells	Biological effects relevant to dermatology
Keratinocytes	miRNAs, cytokines, growth factors	Melanocytes, fibroblasts, immune cells	Regulate pigmentation, barrier signaling, immune modulation
Dermal fibroblasts	Collagen-regulating molecules, MMPs	Keratinocytes, endothelial cells	ECM remodeling, scar formation, wound healing
Melanocytes	Pigment-regulating miRNAs, exosomal melanin	Keratinocytes, immune cells	Melanogenesis and pigment transfer
Mesenchymal stem cells (MSCs)	Anti-inflammatory cytokines, angiogenic factors	Keratinocytes, fibroblasts, macrophages	Wound healing, anti-inflammatory, pro-regenerative
Platelets	PDGF, VEGF, TGF-β	Endothelial cells, fibroblasts	Angiogenesis, tissue repair
Immune cells	Cytokines, antigen-presenting vesicles	T-cells, antigen-presenting cells	Immune regulation, tolerance, or activation

Collectively, these mechanistic insights highlight the central role of exosomes in cutaneous biology and their potential as therapeutic modulators. At the same time, they emphasize that exosomes are not a uniform or interchangeable entity, and their biological effects must be interpreted within the context of source, composition, and target tissue. A rigorous understanding of these mechanisms is essential before extrapolating experimental findings to clinical dermatology practice, particularly in diverse patient populations, such as those encountered in India.

Sources of exosomes used in dermatology and their biological relevance

The biological activity and therapeutic potential of exosomes are intrinsically linked to their cellular source. Differences in parent cell type influence exosomal cargo composition, surface markers, and downstream functional effects. In dermatology, exosomes derived from MSCs, dermal fibroblasts, keratinocytes, platelets, and immune cells have been explored for their regenerative, immunomodulatory, and pigment-modulating properties. Understanding source-specific differences is essential for interpreting experimental data and for rational clinical application.

MSC-Derived Exosomes

MSC-derived exosomes are the most extensively studied in dermatologic research. These vesicles exhibit potent immunomodulatory, pro-angiogenic, and pro-regenerative properties, largely attributed to their enrichment in anti-inflammatory cytokines, growth factors, and regulatory microRNAs. Preclinical models have demonstrated that MSC-derived exosomes promote keratinocyte proliferation, fibroblast migration, angiogenesis, and extracellular matrix remodeling, resulting in accelerated wound healing and improved tissue regeneration [5,20].

In dermatologic contexts, MSC-derived exosomes have been investigated for applications in chronic wounds, acne scarring, photoaging, and hair disorders. Their ability to suppress pro-inflammatory cytokines while enhancing reparative pathways positions them as attractive candidates for inflammatory and regenerative indications. However, heterogeneity in MSC source (bone marrow, adipose tissue, and umbilical cord), isolation protocols, and culture conditions significantly influences exosome composition and biological effects, raising challenges for standardization and reproducibility [8].

Dermal Fibroblast-Derived Exosomes

Dermal fibroblast-derived exosomes play a central role in maintaining dermal homeostasis and mediating epidermal-dermal interactions. These exosomes have been shown to regulate collagen synthesis, fibroblast proliferation, and matrix remodeling, making them particularly relevant to wound healing, scar modulation, and skin aging. Experimental studies suggest that fibroblast-derived exosomes can enhance keratinocyte migration and re-epithelialization, supporting their potential use in regenerative dermatology [21].

Given the pivotal role of fibroblasts in fibrotic and pigmentary disorders, fibroblast-derived exosomes may also influence melanocyte behavior indirectly through modulation of the dermal microenvironment. However, clinical translation remains limited, and its role in therapeutic applications requires further validation.

Keratinocyte-Derived Exosomes

Keratinocyte-derived exosomes contribute significantly to epidermal signaling networks. These vesicles are involved in immune modulation, pigmentation regulation, and barrier homeostasis. Keratinocyte exosomes have been shown to influence melanocyte function by transferring regulatory microRNAs and signaling molecules that affect melanogenesis and pigment distribution [22]. In inflammatory dermatoses, keratinocyte-derived exosomes may amplify or attenuate immune responses, depending on the inflammatory milieu. While these exosomes are of considerable mechanistic interest, their therapeutic exploitation remains largely experimental, with limited direct clinical application at present.

Platelet-Derived Exosomes

Platelet-derived exosomes are enriched in growth factors such as platelet-derived growth factor, TGF-β, and vascular endothelial growth factor. These vesicles have been implicated in angiogenesis, wound healing, and tissue repair. Given the widespread use of platelet-rich plasma in dermatology, platelet-derived exosomes have attracted attention as a potential mediator of PRP’s biological effects [23]. However, platelet-derived exosomes also carry pro-coagulant and pro-inflammatory components, necessitating careful evaluation of safety and consistency. Their therapeutic role remains incompletely defined, particularly in chronic inflammatory skin diseases.

Immune Cell-Derived Exosomes

Exosomes derived from immune cells such as macrophages, dendritic cells, and T lymphocytes play a role in antigen presentation and immune regulation within the skin. These vesicles can influence T-cell polarization, cytokine production, and local immune responses, contributing to the pathogenesis of inflammatory and autoimmune dermatoses [24].

While immune cell-derived exosomes offer intriguing therapeutic possibilities, particularly for immune-mediated skin diseases, their complexity and potential for off-target immune effects pose significant translational challenges.

Relevance to Indian Dermatology Practice

In Indian clinical practice, most commercially available exosome-based products are marketed as “MSC-derived,” often without transparent disclosure of cellular source, isolation methods, or quality-control parameters. This lack of standardization complicates the interpretation of clinical outcomes and raises concerns regarding consistency, safety, and ethical use. Given the diversity of exosome sources and their distinct biological profiles, clinicians must exercise caution in extrapolating preclinical findings to real-world practice, particularly in the absence of robust regulatory oversight.

Dermatologic indications: critical appraisal of evidence

Despite growing clinical interest, the evidence supporting exosome-based therapies in dermatology remains heterogeneous, with a predominance of preclinical and early-phase human studies. The strength of evidence varies considerably across indications, and careful appraisal is essential before extrapolating findings to routine clinical practice, particularly in the Indian context.

Pigmentary Disorders

Pigmentary disorders such as melasma and post-inflammatory hyperpigmentation represent a major therapeutic challenge in Indian patients. Exosomes have been proposed as potential modulators of pigmentation through their effects on melanocyte activity, melanosome transfer, and inflammatory signaling. Preclinical studies suggest that exosomes derived from MSCs and keratinocytes can downregulate tyrosinase activity and suppress melanogenesis via microRNA-mediated pathways, including modulation of MITF expression [24,25].

Limited clinical studies have explored the use of exosome-containing formulations for pigmentary conditions, primarily in small, uncontrolled cohorts. Some reports describe improvement in pigmentation and skin tone, particularly when exosomes are combined with energy-based devices or microneedling. However, variability in study design, exosome source, outcome measures, and follow-up duration precludes definitive conclusions. Importantly, robust randomized controlled trials evaluating efficacy and safety in darker skin types are lacking, raising concerns regarding reproducibility and long-term outcomes in Indian populations [15].

Acne Scars and Wound Healing

The regenerative potential of exosomes has been most consistently demonstrated in wound healing and scar modulation models. Preclinical studies indicate that exosomes enhance fibroblast migration, angiogenesis, and extracellular matrix remodeling, leading to improved wound closure and reduced fibrosis. These effects are mediated through activation of signaling pathways such as PI3K/Akt and TGF-β modulation [26,27].

Early clinical studies and case series have reported improvement in acne scars and skin texture following exosome-based interventions, often used as adjuncts to laser resurfacing or microneedling. While these findings are encouraging, most studies lack control groups and standardized outcome assessment. Additionally, the contribution of exosomes relative to procedural interventions remains unclear. In Indian practice, where acne scarring is prevalent and post-inflammatory hyperpigmentation is a significant concern, cautious interpretation of these results is warranted [28].

Hair Disorders

Hair loss disorders, including androgenetic alopecia and alopecia areata, have emerged as potential targets for exosome-based therapies. Exosomes derived from dermal papilla cells and MSCs have been shown to promote hair follicle cycling, prolong the anagen phase, and enhance hair shaft production in experimental models. These effects are attributed to activation of Wnt/β-catenin signaling and modulation of inflammatory pathways within the hair follicle microenvironment [11,29].

Clinical evidence in hair disorders remains preliminary, consisting largely of small-scale studies and observational reports. Some studies have demonstrated increased hair density and thickness following exosome-based treatments, often in combination with microneedling. However, standardized protocols, long-term efficacy data, and comparative studies with established therapies are lacking. In the Indian context, where hair loss carries significant psychosocial impact, these therapies should currently be regarded as investigational rather than established treatments [19].

Inflammatory Dermatoses

The immunomodulatory properties of exosomes have generated interest in their potential role in inflammatory skin diseases, such as atopic dermatitis and psoriasis. Preclinical studies suggest that exosomes can suppress pro-inflammatory cytokine production, promote regulatory immune responses, and restore barrier function. However, translation of these findings into clinical practice has been limited.

Clinical data supporting the use of exosomes in inflammatory dermatoses are sparse and largely anecdotal. Given the availability of well-validated systemic and biologic therapies, with established efficacy and safety profiles, the role of exosome-based interventions in inflammatory skin diseases remains uncertain. In India, where infection risk and long-term safety considerations are paramount, the adoption of exosome therapies for inflammatory dermatoses without robust evidence warrants particular caution [30].

Summary of Evidence and Indian Relevance

Overall, the current evidence base for exosome-based therapies in dermatology is characterized by strong mechanistic rationale and promising preclinical data, but limited high-quality clinical evidence. The gap between experimental promise and clinical validation is especially relevant in India, where patient expectations, commercial pressures, and regulatory ambiguity intersect.

From an evidence-based perspective, exosome-based interventions should be considered investigational across most dermatologic indications. Well-designed randomized controlled trials, particularly involving Indian patients and skin of color, are urgently needed to establish efficacy, safety, optimal dosing, and long-term outcomes [31].

Exosomes in the skin of color and relevance to Indian patients

Skin of color exhibits distinct structural, functional, and immunologic characteristics that influence disease presentation, therapeutic response, and risk of adverse effects. Indian patients, predominantly represented by Fitzpatrick skin types IV-VI, demonstrate heightened melanocyte activity, increased melanosome size and dispersion, and distinct inflammatory responses compared with lighter skin types. These differences have important implications for the translational application of exosome-based therapies in dermatology.

Biological Considerations in Skin of Color

Melanocyte-keratinocyte interactions are central to pigmentation homeostasis and are tightly regulated by paracrine signaling, cytokine gradients, and microRNA-mediated pathways. Exosomes have been shown to participate in this regulatory network by transferring melanogenesis-modulating cargo, including microRNAs that influence tyrosinase activity, melanosome transport, and MITF expression. In skin of color, where baseline melanogenic activity is higher, modulation of these pathways may produce exaggerated or unpredictable effects if not carefully controlled [32,33].

Furthermore, inflammatory responses in skin of color tend to be more persistent and pigment-altering, contributing to a higher incidence of post-inflammatory hyperpigmentation following acne, eczema, procedures, or trauma. Exosomes capable of modulating inflammatory signaling and wound repair pathways may theoretically reduce pigmentary sequelae; however, robust evidence demonstrating consistent benefit in darker skin types is currently lacking.

Implications for Pigmentary Disorders

Pigmentary disorders such as melasma and post-inflammatory hyperpigmentation are disproportionately prevalent in Indian populations and represent a major therapeutic challenge; in a multicity Indian study of 1204 women, the prevalence of melasma ranged from ~15% to ~28% across sites [33]. While preclinical data suggest that exosomes may suppress melanogenesis and promote pigment normalization, the absence of high-quality clinical trials specifically enrolling patients with skin of color limits confidence in these findings.

Importantly, interventions that alter melanocyte signaling carry the risk of paradoxical hyperpigmentation or hypopigmentation, particularly when combined with procedural therapies. In Indian patients, where pigmentary stability is a key treatment goal, unvalidated exosome-based interventions may inadvertently exacerbate dyschromia if not applied judiciously [34].

Wound Healing, Scarring, and Fibrosis

Skin of color demonstrates a greater propensity for hypertrophic scarring and keloid formation, reflecting differences in fibroblast activity, collagen deposition, and inflammatory signaling. Exosomes derived from MSCs and fibroblasts have been shown to modulate fibroblast proliferation and extracellular matrix remodeling in experimental models. While these effects suggest potential utility in scar modulation, their impact on aberrant fibrosis in keloid-prone skin remains uncertain [35].

Given the high prevalence of acne scarring and post-procedural pigmentation in Indian patients, the application of exosome-based therapies as adjuncts to energy-based or minimally invasive procedures requires careful evaluation. Evidence supporting their ability to reduce scarring or pigmentary complications in skin of color is currently insufficient to support routine use.

Hair Disorders in Indian Populations

Hair loss disorders, including androgenetic alopecia and alopecia areata, are common in Indian patients and often associated with significant psychosocial distress. While exosome-based approaches have shown promise in experimental models of hair follicle regeneration, clinical data in Indian populations are sparse. Variations in hair follicle biology, scalp inflammation, and genetic predisposition may influence therapeutic response, underscoring the need for population-specific studies [36].

Safety Considerations and Long-Term Outcomes

A critical concern in the application of exosome-based therapies in skin of color is the paucity of long-term safety data. Alterations in pigmentation pathways, immune signaling, or fibroblast activity may have delayed consequences that are not captured in short-term studies. In Indian patients, where pigmentary alterations can have a profound cosmetic and psychological impact, the absence of longitudinal safety data represents a significant barrier to widespread clinical adoption [37].

Summary and Indian Context

In summary, while exosome-based therapies hold theoretical promise for addressing conditions prevalent in Indian dermatology practice, their application in skin of color demands particular caution. Differences in pigmentation biology, inflammatory responses, and scarring tendencies necessitate rigorous, population-specific evaluation. Until high-quality clinical trials involving Indian patients are conducted, exosome-based interventions should be considered investigational, and their use should be guided by ethical practice principles and transparent patient counseling.

Clinical practice trends in India - promise versus reality

Over the past five years, exosome-based interventions have entered Indian dermatology practice, primarily through the aesthetic and regenerative medicine sectors. These therapies are frequently marketed as “stem-cell derived,” “cell-free regenerative,” or “next-generation biologics,” and are commonly used as adjuncts to microneedling, laser resurfacing, hair regeneration procedures, or topical rejuvenation therapies. However, the pace of commercialization has far exceeded the generation of robust clinical evidence or regulatory clarity, creating a landscape characterized by scientific promise but significant uncertainty (Table 2).

**Table 2:** Practical considerations for Indian clinical practice PIH: post-inflammatory hyperpigmentation

Domain	Key considerations
Skin of color	Higher PIH risk; pigment modulation must be cautious
Scarring tendency	Higher risk of keloid/hypertrophic scars
Infection burden	Screen quality & sterility of biologicals
Regulatory ambiguity	Many products not regulated as drugs
Consent	Must clearly label “investigational”
Cost burden	Typically self-paid; cost-value must be disclosed
Patient expectation	High - influenced by social media marketing

Commercialization Ahead of Regulation

In India, many exosome-containing formulations are introduced under the classification of cosmeceuticals or aesthetic biologics, rather than as regulated pharmaceutical products. As a result, these products may enter the market without a comprehensive characterization of exosome content, source validation, sterility assurance, or potency testing. International societies have emphasized the need for stringent manufacturing standards - including source cell characterization, isolation methodology, cargo profiling, and batch-to-batch consistency - to ensure safety and reproducibility [38]. However, real-world practice in India often reflects variable adherence to such standards.

The absence of a unified regulatory pathway for exosome-based products creates ambiguity for clinicians and patients alike. Products may be advertised as “stem-cell derived,” despite containing undefined EV fractions, protein lysates, or conditioned media, rather than purified exosomes. This lack of transparency complicates informed consent, blurs the boundaries between innovation and experimentation, and exposes clinicians to medico-legal vulnerability [37].

From a policy perspective, a clearer regulatory pathway for exosome/EV products is needed, including explicit product classification and minimum manufacturing and evidence standards. Regulators could require standardized nomenclature and restrict “stem-cell derived” marketing claims unless source, content, and dose are verifiable. Minimum release criteria should include characterization of identity (particle size distribution and EV markers), quantification and dose metrics, purity (to distinguish purified EV/exosome preparations from conditioned media or protein lysates), potency assays linked to mechanism of action, sterility/endotoxin testing, stability, and batch-to-batch consistency. In parallel, labeling and advertising should be aligned with demonstrated composition and supported indications, with stepwise clinical evidence requirements and robust post-marketing pharmacovigilance and traceability (lot/batch) to protect patients and guide clinicians.

Variability in Clinical Protocols

Clinical use of exosomes in India is characterized by considerable heterogeneity in treatment protocols. Variations exist in product source (e.g., MSC-derived, fibroblast-derived, platelet-derived), delivery route (topical, intradermal, microneedling-assisted), dosing intervals, and combination strategies with energy-based devices. Few protocols are standardized, or supported by strong clinical trial data, and outcome assessment is often subjective.

In cosmetic practice, exosomes are frequently positioned as adjunctive enhancers of procedural outcomes for acne scars, skin rejuvenation, or hair growth. While anecdotal reports and uncontrolled case series suggest improvement in texture, pigmentation, and hair density, the contribution of exosomes relative to the procedural intervention itself remains unclear. This ambiguity is particularly relevant in Indian patients, in whom post-inflammatory hyperpigmentation and scarring risk necessitate cautious escalation of procedural intensity [28].

Patient Expectations and Ethical Responsibilities

High patient demand for regenerative and “natural” therapies, amplified by social media marketing, has fueled the rapid adoption of exosome-based interventions in India. Many patients perceive exosomes as safer, or more “biological,” alternatives to pharmaceuticals or injectables. In this context, dermatologists shoulder a heightened ethical responsibility to ensure accurate communication regarding the investigational nature of such therapies, the current level of evidence, and the uncertainties surrounding long-term safety.

Ethical practice requires robust informed consent that clearly distinguishes between established therapies and experimental interventions. International guidance frameworks have emphasized the importance of avoiding exaggerated claims, maintaining scientific transparency, and ensuring that innovation does not outpace ethical governance [39]. These principles are especially salient in India, where variable literacy levels, social desirability pressures, and direct-to-consumer marketing may influence decision-making.

Cost, Access, and Equity

Exosome-based treatments are typically high-cost, out-of-pocket services in India. Given the limited availability of high-quality clinical data, the ethical justification for expensive interventions must be carefully weighed against uncertain benefits. There is also a risk that unproven regenerative therapies may disproportionately target affluent, urban populations, potentially widening disparities in access to validated dermatologic care.

Gap Between Promise and Practice

Taken together, clinical practice trends in India reveal a substantial gap between the mechanistic promise of exosomes and the strength of current clinical evidence. While early data are encouraging and justify continued scientific exploration, widespread real-world use has outpaced regulatory frameworks, quality-control mechanisms, and long-term safety monitoring. Until high-quality randomized controlled trials - ideally including Indian patients and skin-of-color cohorts - provide clearer guidance, exosome-based therapies should be considered investigational and used with caution, transparency, and strong ethical safeguards.

Regulatory, quality-control, and medico-legal challenges in India

**Figure 3:**
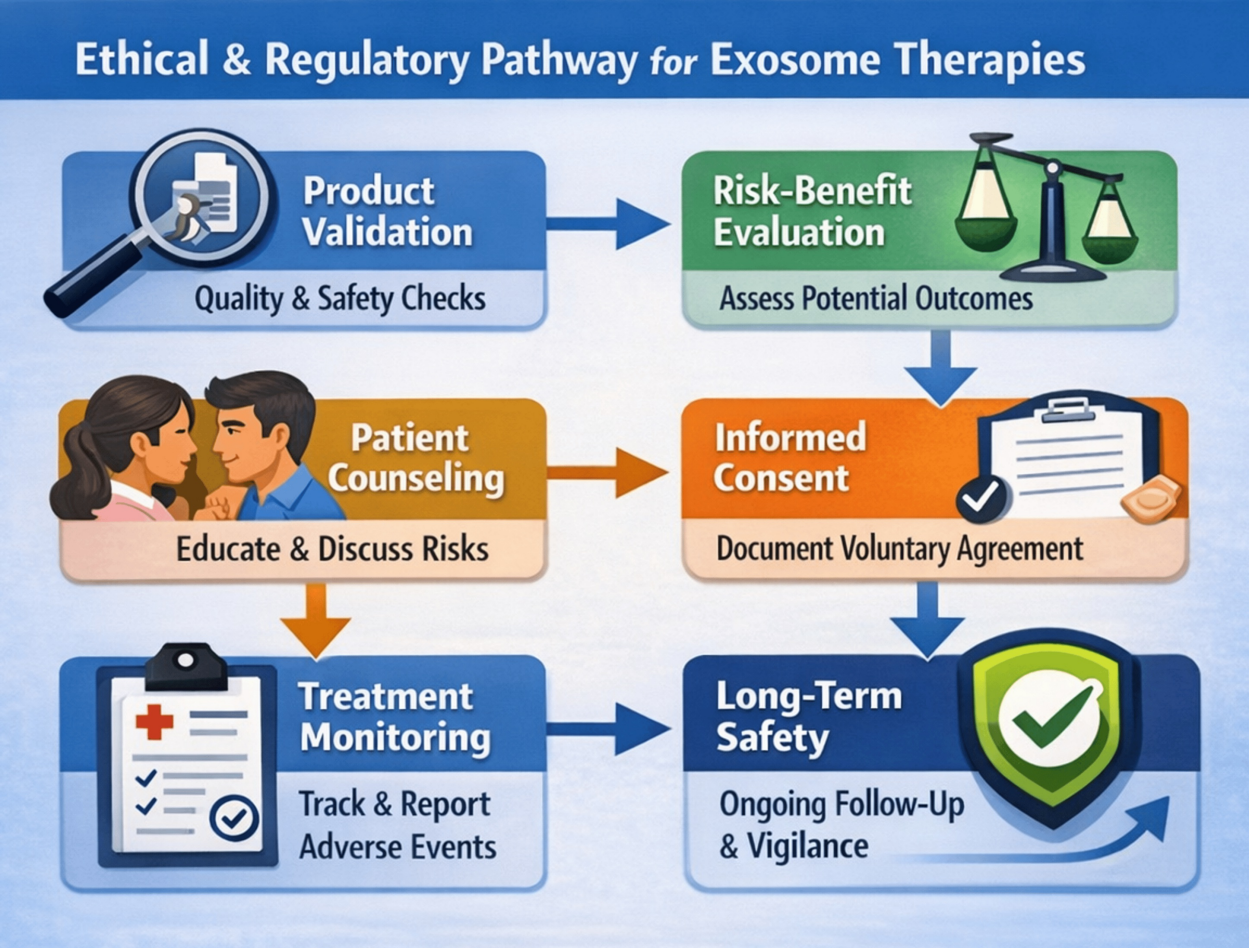
Ethical and regulatory pathway for responsible integration of exosome-based therapies Suggested clinical governance framework supporting safe introduction of exosome-based dermatologic therapies. Key elements include verification of product quality and source; structured risk-benefit evaluation; comprehensive patient counseling; robust informed-consent documentation; systematic monitoring and reporting of treatment outcomes and adverse events; and ongoing long-term safety surveillance. This is an original illustration.

The rapid introduction of exosome-based products into aesthetic and regenerative dermatology in India has occurred within a regulatory environment that is still evolving. Exosomes occupy a grey zone between biologics, cell-derived products, and advanced therapeutic medicinal products. As a result, regulatory oversight, quality-control enforcement, and medico-legal frameworks remain variably defined, posing challenges for clinicians, manufacturers, and patients (Figure 3).

Regulatory Classification Ambiguity

Indian regulatory systems - including the Central Drugs Standard Control Organisation (CDSCO) and the Indian Council of Medical Research (ICMR) - have established governance for stem-cell-based interventions and new drugs; however, cell-free exosomes are not yet uniformly categorized. In many cases, exosome-containing formulations are marketed as cosmeceuticals, biological preparations, or research-grade products, thereby bypassing the level of scrutiny typically applied to pharmaceuticals or biological drugs. Internationally, exosome-based therapeutics are increasingly being considered under frameworks used for advanced biological products, with calls for rigorous manufacturing and characterization standards before clinical use [40].

In India, the absence of explicit classification risks the entry of heterogeneous products into clinical practice, some of which may contain undefined EV fractions, protein lysates, or conditioned media rather than purified exosomes. This ambiguity creates uncertainty about regulatory accountability, post-marketing surveillance mechanisms, and legal responsibility in the event of adverse outcomes.

Quality-Control and Manufacturing Variability

Uniform characterization of exosome products remains one of the greatest challenges worldwide. The International Society for Extracellular Vesicles (ISEV) has emphasized that exosome-based therapeutics require robust quality standards, including: identification of source cell lines, standardized isolation and purification techniques, sterility and endotoxin testing, defined potency assays, and batch-to-batch consistency to ensure reproducibility and safety [41].

In practice, many commercially available products marketed in India provide limited information on vesicle composition, RNA and protein cargo, particle size distribution, or purity. Cold-chain integrity, contamination risks, residual cell-derived material, and immunogenic potential may vary depending on the manufacturing process. Without enforceable quality-control standards and independent verification, clinicians cannot reliably determine the identity, purity, or biological behavior of exosome products used in routine practice [42].

Informed Consent and Medico-Legal Accountability

The medico-legal implications of using exosome-based therapies in India are significant. In the absence of explicit regulatory approval for specific dermatologic indications, many clinical uses fall within the domain of off-label or experimental therapy. Ethical practice standards require that patients be clearly informed of: the investigational nature of therapy, the level of available evidence, potential risks and uncertainties, and alternative validated treatment options before consenting [43].

Failure to obtain robust informed consent exposes clinicians to negligence or misrepresentation claims, particularly if outcomes are suboptimal or complications arise. Furthermore, misleading advertising or unsubstantiated claims, especially through digital and social media platforms, may constitute ethical and legal violations under consumer protection and medical advertising regulations [44].

Need for Regulatory Convergence and Surveillance

India’s regulatory framework for biologics and cell-based therapies is progressively strengthening; however, exosome-specific policy guidance is needed to ensure safe and ethical translation. Development of national standards aligned with international guidance on EV therapeutics - including classification, manufacturing controls, labeling, and pharmacovigilance - would support both innovation and patient safety.

Equally important is the establishment of structured adverse-event reporting systems and clinical registries to monitor long-term safety and treatment outcomes. Such measures would help ensure that clinical use progresses in parallel with scientific understanding rather than ahead of it [45]. In summary, the regulatory and medico-legal environment surrounding exosome-based dermatologic therapies in India remains nascent. Ambiguity in classification, variability in product quality, and gaps in informed consent processes create potential risk for both clinicians and patients. Clear national policy, rigorous manufacturing oversight, and ethically grounded clinical practice are essential to safeguard patient welfare while enabling responsible innovation.

Future directions, Indian research priorities, and conclusion

Future Directions in Exosome Science and Therapeutics

The next phase of exosome translation in dermatology will be defined by standardization, precision engineering, and rigorous clinical validation. Advances in bioengineering now enable cargo-modification, surface functionalization, and targeted delivery, allowing the development of exosomes enriched in selected microRNAs, growth factors, or immunoregulatory molecules for specific dermatologic indications [46]. Parallel progress in nanotechnology and analytical chemistry is improving our ability to characterize vesicle populations with higher resolution, facilitating clearer differentiation between exosomes, microvesicles, and other EV subtypes.

Future therapeutic strategies are likely to evolve from heterogeneous, biologically derived vesicles toward well-defined, reproducible products manufactured under Good Manufacturing Practice (GMP) conditions. These developments will be crucial for regulatory acceptance, pharmacovigilance, and clinician confidence.

Indian Research and Policy Priorities

For India, the imperative is two-fold: generate high-quality clinical evidence while establishing robust governance frameworks. Indian research priorities should include: (i) randomized controlled trials evaluating efficacy and safety across pigmentary disorders, acne scars, hair disorders, and wound healing - with deliberate inclusion of skin-of-color cohorts; (ii) pharmacokinetic and biodistribution studies to determine optimal dosing, delivery routes, and tissue penetration in human skin; (iii) long-term safety registries to monitor pigmentary change, fibrosis, immune effects, and delayed adverse outcomes; (iv) head-to-head comparisons with established therapies to define true incremental benefit; (v) cost-effectiveness analyses relevant to the Indian healthcare ecosystem.

Equally critical is policy clarity. Development of India-specific guidance aligned with international EV standards would help ensure quality assurance, ethical transparency, and patient protection [47]. Engagement between clinicians, academic institutions, regulatory bodies, and industry partners will be essential to build an ecosystem that fosters innovation while safeguarding public trust.

Responsible Clinical Integration

Until such evidence and policy scaffolding mature, exosome-based dermatologic therapies should be regarded as investigational in India. Ethical clinical integration requires: transparent patient counseling, explicit informed consent, avoidance of exaggerated claims, prioritization of evidence-based alternatives, and documentation of outcomes and adverse events. Such measures align with the emerging global consensus that regenerative therapies must not outpace the science that justifies them.

## Conclusions

Exosomes represent one of the most intriguing frontiers in contemporary dermatology, offering a biologically elegant means of modulating inflammation, pigmentation, wound repair, and hair follicle biology. Their appeal is particularly strong in India, where pigmentary disorders, scarring, and hair loss are prevalent, chronic, and often refractory to conventional therapies. Yet, the scientific promise of exosomes currently exceeds the strength of clinical evidence, and regulatory, quality-control, and long-term safety frameworks remain incompletely defined.

A rigorous, ethically grounded, and India-specific approach is required to bridge this gap. By prioritizing methodologically robust research, transparent regulation, and patient-centered practice, the dermatology community in India can help shape the responsible evolution of exosome-based therapeutics, ensuring that innovation serves patient welfare rather than commercial momentum.

## References

[1] Kalluri R, LeBleu VS (2020). The biology, function, and biomedical applications of exosomes. Science.

[2] Raposo G, Stoorvogel W (2013). Extracellular vesicles: exosomes, microvesicles, and friends. J Cell Biol.

[3] Valadi H, Ekström K, Bossios A, Sjöstrand M, Lee JJ, Lötvall JO (2007). Exosome-mediated transfer of mRNAs and microRNAs is a novel mechanism of genetic exchange between cells. Nat Cell Biol.

[4] Kim H, Lee JW, Han G, Kim K, Yang Y, Kim SH (2021). Extracellular vesicles as potential theranostic platforms for skin diseases and aging. Pharmaceutics.

[5] Hu L, Wang J, Zhou X (2016). Exosomes derived from human adipose mensenchymal stem cells accelerates cutaneous wound healing via optimizing the characteristics of fibroblasts. Sci Rep.

[6] Zhang B, Wu X, Zhang X (2015). Human umbilical cord mesenchymal stem cell exosomes enhance angiogenesis through the Wnt4/β-catenin pathway. Stem Cells Transl Med.

[7] Kim YJ, Yoo SM, Park HH (2017). Exosomes derived from human umbilical cord blood mesenchymal stem cells stimulates rejuvenation of human skin. Biochem Biophys Res Commun.

[8] Phinney DG, Pittenger MF (2017). Concise review: MSC-derived exosomes for cell-free therapy. Stem Cells.

[9] Rahman E, Carruthers JD, Rao P (2025). Regenerative aesthetics: a genuine frontier or just a facet of regenerative medicine: a systematic review. Aesthetic Plast Surg.

[10] Alexis AF, Woolery-Lloyd H, Williams K (2021). Racial/ethnic variations in skin barrier: implications for skin care recommendations in skin of color. J Drugs Dermatol.

[11] Lener T, Gimona M, Aigner L (2015). Applying extracellular vesicles based therapeutics in clinical trials - an ISEV position paper. J Extracell Vesicles.

[12] Colombo M, Raposo G, Théry C (2014). Biogenesis, secretion, and intercellular interactions of exosomes and other extracellular vesicles. Annu Rev Cell Dev Biol.

[13] Chen L, Chen R, Kemper S, Brigstock DR (2018). Pathways of production and delivery of hepatocyte exosomes. J Cell Commun Signal.

[14] Huang P, Bi J, Owen GR (2015). Keratinocyte microvesicles regulate the expression of multiple genes in dermal fibroblasts. J Invest Dermatol.

[15] Bellei B, Picardo M (2020). Premature cell senescence in human skin: dual face in chronic acquired pigmentary disorders. Ageing Res Rev.

[16] Yang Y, Huang Y, Yang J (2025). Umbilical cord mesenchymal stem cell-derived exosomes promote wound healing and skin regeneration via the regulation of inflammation and angiogenesis. Front Bioeng Biotechnol.

[17] Yan X, Guo YX, Liu YX, Liu C (2025). Mesenchymal stem cell-derived exosomes and the Wnt/β-catenin pathway: unifying mechanisms of multi-organ regeneration and the path to precision clinical translation. World J Stem Cells.

[18] Robbins PD, Morelli AE (2014). Regulation of immune responses by extracellular vesicles. Nat Rev Immunol.

[19] Théry C, Witwer KW, Aikawa E (2018). Minimal information for studies of extracellular vesicles 2018 (MISEV2018): a position statement of the International Society for Extracellular Vesicles and update of the MISEV2014 guidelines. J Extracell Vesicles.

[20] Zhang B, Wang M, Gong A (2015). HucMSC-exosome mediated Wnt4 signaling is required for cutaneous wound healing. Stem Cells.

[21] Lv H, Liu H, Sun T, Wang H, Zhang X, Xu W (2022). Exosome derived from stem cell: a promising therapeutics for wound healing. Front Pharmacol.

[22] Lo Cicero A, Stahl PD, Raposo G (2015). Extracellular vesicles shuffling intercellular messages: for good or for bad. Curr Opin Cell Biol.

[23] Italiano JE Jr, Mairuhu AT, Flaumenhaft R (2010). Clinical relevance of microparticles from platelets and megakaryocytes. Curr Opin Hematol.

[24] Wang X, Cui Z, Zeng B, Qiong Z, Long Z (2022). Human mesenchymal stem cell derived exosomes inhibit the survival of human melanoma cells through modulating miR-138-5p/SOX4 pathway. Cancer Biomark.

[25] Hushcha Y, Blo I, Oton-Gonzalez L, Mauro GD, Martini F, Tognon M, Mattei M (2021). microRNAs in the regulation of melanogenesis. Int J Mol Sci.

[26] Zhang J, Guan J, Niu X (2015). Exosomes released from human induced pluripotent stem cells-derived MSCs facilitate cutaneous wound healing by promoting collagen synthesis and angiogenesis. J Transl Med.

[27] Wang L, Hu L, Zhou X (2021). Author correction: exosomes secreted by human adipose mesenchymal stem cells promote scarless cutaneous repair by regulating extracellular matrix remodelling. Sci Rep.

[28] Shah M, Dukharan V, Broughton L, Stegura C, Schur N, Samman L, Schlesinger T (2025). Exosomes for aesthetic dermatology: a comprehensive literature review and update. J Cosmet Dermatol.

[29] Kwack MH, Seo CH, Gangadaran P, Ahn BC, Kim MK, Kim JC, Sung YK (2019). Exosomes derived from human dermal papilla cells promote hair growth in cultured human hair follicles and augment the hair-inductive capacity of cultured dermal papilla spheres. Exp Dermatol.

[30] Rajendran RL, Gangadaran P, Seo CH (2020). Macrophage-derived extracellular vesicle promotes hair growth. Cells.

[31] Poddar N, Aratikatla A, Gupta A (2025). Therapeutic potential of stem cell-derived exosomes in hair regeneration: a systematic review. World J Stem Cells.

[32] Tadokoro T, Yamaguchi Y, Batzer J (2005). Mechanisms of skin tanning in different racial/ethnic groups in response to ultraviolet radiation. J Invest Dermatol.

[33] Hourblin V, Nouveau S, Roy N, de Lacharrière O (2014). Skin complexion and pigmentary disorders in facial skin of 1204 women in 4 Indian cities. Indian J Dermatol Venereol Leprol.

[34] Callender VD, St Surin-Lord S, Davis EC, Maclin M (2011). Postinflammatory hyperpigmentation: etiologic and therapeutic considerations. Am J Clin Dermatol.

[35] Ogawa R (2017). Keloid and hypertrophic scars are the result of chronic inflammation in the reticular dermis. Int J Mol Sci.

[36] Trüeb RM (2002). Molecular mechanisms of androgenetic alopecia. Exp Gerontol.

[37] Sipp D, Caulfield T, Kaye J (2017). Marketing of unproven stem cell-based interventions: a call to action. Sci Transl Med.

[38] Daley GQ, Hyun I, Apperley JF (2016). Setting global standards for stem cell research and clinical translation: the 2016 ISSCR guidelines. Stem Cell Reports.

[39] Li Q, Li Y, Shao J (2025). Exploring regulatory frameworks for exosome therapy: insights and perspectives. Health Care Sci.

[40] Indian Council of Medical Research (2017). National Ethical Guidelines for Biomedical and Health Research Involving Human Participants.

[41] Witwer KW, Van Balkom BW, Bruno S (2019). Defining mesenchymal stromal cell (MSC)-derived small extracellular vesicles for therapeutic applications. J Extracell Vesicles.

[42] Lötvall J, Hill AF, Hochberg F (2014). Minimal experimental requirements for definition of extracellular vesicles and their functions: a position statement from the International Society for Extracellular Vesicles. J Extracell Vesicles.

[43] Sacchidanand SA, Bhat S (2012). Safe practice of cosmetic dermatology: avoiding legal tangles. J Cutan Aesthet Surg.

[44] György B, Hung ME, Breakefield XO, Leonard JN (2015). Therapeutic applications of extracellular vesicles: clinical promise and open questions. Annu Rev Pharmacol Toxicol.

[45] Elsharkasy OM, Nordin JZ, Hagey DW, de Jong OG, Schiffelers RM, Andaloussi SE, Vader P (2020). Extracellular vesicles as drug delivery systems: why and how?. Adv Drug Deliv Rev.

[46] Riau AK, Ong HS, Yam GH, Mehta JS (2019). Sustained delivery system for stem cell-derived exosomes. Front Pharmacol.

[47] Sipp D, Robey PG, Turner L (2018). Clear up this stem-cell mess. Nature.

